# Brilliant Ideas Can Come in All Sizes: Research Letters

**DOI:** 10.2196/41046

**Published:** 2022-07-26

**Authors:** Rita Kukafka, Tiffany I Leung, Gunther Eysenbach

**Affiliations:** 1 Columbia University Irving Medical Center New York, NY United States; 2 JMIR Publications, Inc Toronto, ON Canada; 3 University of Victoria Victoria, BC Canada

**Keywords:** open science, open access publishing, publishing, scholarly publishing, scientific publishing, research, scientific research, research letter

## Abstract

The *Journal of Medical Internet Research* is pleased to offer “Research Letter” as a new article type. Research Letters are similar to original and short paper types in that they report the original results of studies in a peer-reviewed, structured scientific communication. The Research Letter article type is optimal for presenting new, early, or sometimes preliminary research findings, including interesting observations from ongoing research with significant implications that justify concise and rapid communication.

Did you know that Albert Einstein published his famous E=mc² equation on mass-energy equivalence in roughly 2 pages [1]? Or that the original and preliminary communication suggesting the double-helix structure of DNA by Watson and Crick (Figure 1 [2,3]) is also only a little more than 1 page in length? If winning a Nobel prize is evidence of brilliance, then one may conclude that the length of a manuscript is not commensurate with its value.

Because less is sometimes more, the *Journal of Medical Internet Research* is now pleased to offer “Research Letter” as a new article type. Research Letters in the *Journal of Medical Internet Research* are similar to original and short paper types in that they report the original results of studies in a peer-reviewed, structured scientific communication. The Research Letter article type is optimal for presenting new, early, or sometimes preliminary research findings, including interesting observations from ongoing research with significant implications that justify concise and rapid communication.

The *Journal of Medical Internet Research* is publishing Research Letters for several reasons. First, the Research Letter is an optimal medium for quickly communicating transformative work, offering authors an opportunity to submit their focused research work for potentially more rapid peer review and publication processes simply by the nature of the communication. Second, larger and more extensive research on contemporary issues might also produce focused findings that may be incidental to the primary aims, yet still be valuable to report. One interesting key result can be displayed in 1 or 2 tables or figures. Additionally, students and early career researchers are encouraged to submit Research Letters as a pathway for reporting their impactful, targeted research projects; this may offer a stepping stone for these researchers as they publish work that contributes to the field and to their scientific growth and professional advancement. For readers, who often include busy scientists and professionals, Research Letters can offer new ideas or approaches in a brief and quickly digestible, yet robust and high-quality, manner. Taking experiences from other high-impact journals, Research Letters are often highly cited.

**Figure 1:**
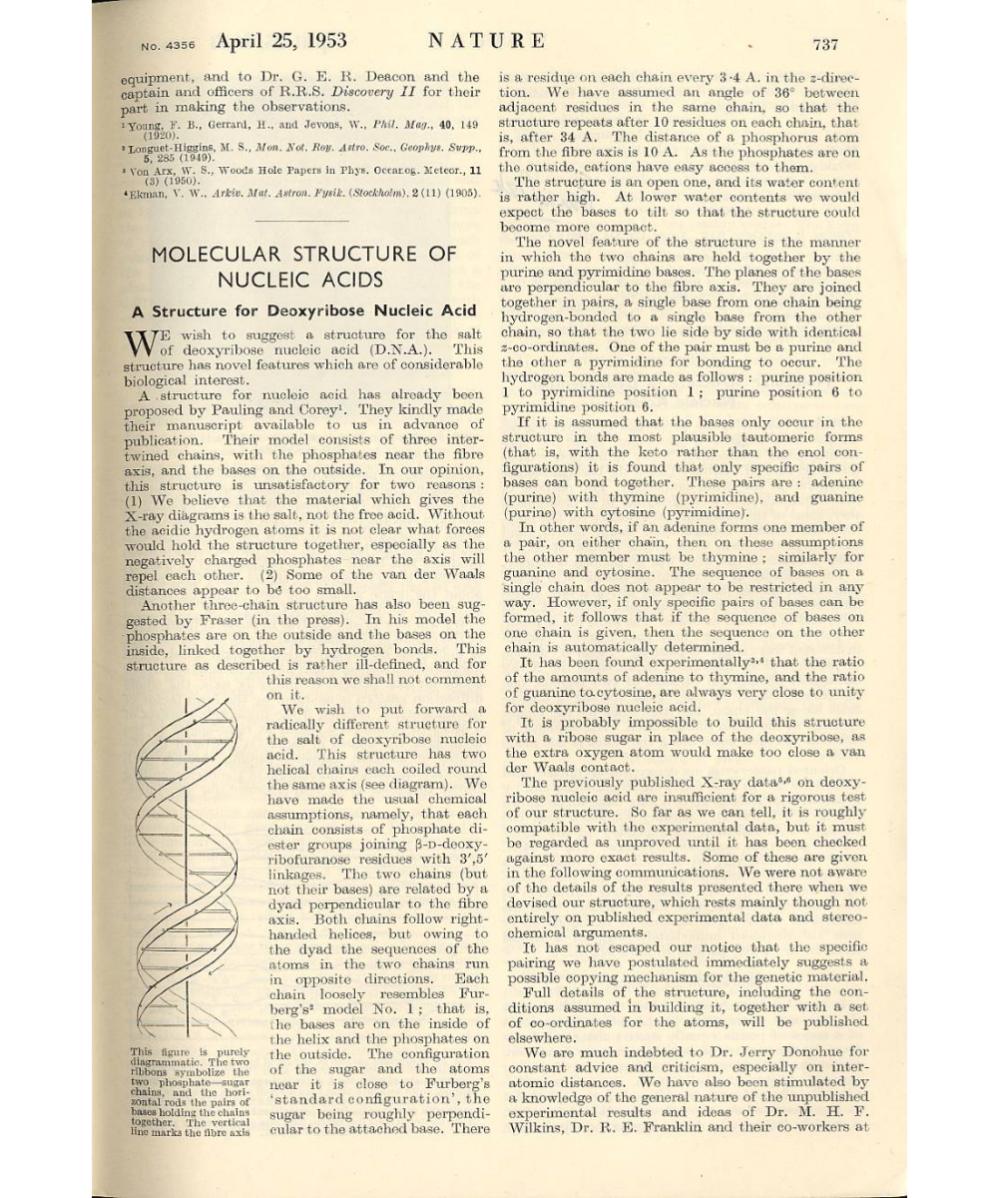
Archived scan of "Molecular Structure of Nucleic Acids: A Structure for Deoxyribose Nucleic Acid," published on April 25, 1953, by Watson and Crick [2]. Source: Linus Pauling and the Race for DNA [3].

Research Letters should still present original work that has not been previously published. Work presented at a conference that has not been previously published in proceedings can be submitted as a Research Letter. However, tables or figures from previously published or submitted papers would not be considered in a Research Letter. Authors can refer to article type information on the format of a Research Letter in JMIR Publication’s Knowledge Base [4]. In this issue of the *Journal of Medical Internet Research*, the journal has published its first example [5], with additional Research Letters currently in review.

We encourage authors to consider submitting their Research Letters to the *Journal of Medical Internet Research*. Additionally, the journal editors may suggest to authors the Research Letter article type as a more suitable format for their work. This is not intended to undersell the contribution of the submission. Authors may not realize that the Research Letter is subject to the same rigorous peer-review process as other article types here at JMIR Publications. As we have seen from Einstein and other eminent Nobel Prize winners, brilliant ideas can be expressed succinctly.

We look forward to reviewing and publishing your Research Letters!
